# A nomogram for predicting atrial fibrillation detected after acute ischemic stroke

**DOI:** 10.3389/fneur.2022.1005885

**Published:** 2022-10-14

**Authors:** Ming Pang, Zhuanyun Li, Lin Sun, Na Zhao, Lina Hao

**Affiliations:** ^1^Neuroelectrophysiology Room, Function Department, Cangzhou Hospital of Integrated Traditional Chinese Medicine and Western Medicine, Cangzhou, China; ^2^Department of Emergency Medicine, Union Hospital of Tongji Medical College, Huazhong University of Science and Technology, Wuhan, China; ^3^Department of Neurology, Cangzhou Hospital of Integrated Traditional Chinese Medicine and Western Medicine, Cangzhou, China

**Keywords:** acute ischemic stroke, atrial fibrillation, risk factors, prediction model, nomogram

## Abstract

**Background:**

Atrial fibrillation detected after stroke (AFDAS) is associated with an increased risk of ischemic stroke (IS) recurrence and death. Early diagnosis can help identify strategies for secondary prevention and improve prognosis. However, there are no validated predictive tools to assess the population at risk for AFDAS. Therefore, this study aimed to develop and validate a predictive model for assessing the incidence of AFDAS after acute ischemic stroke (AIS).

**Methods:**

This study was a multicenter retrospective study. We collected clinical data from 5332 patients with AIS at two hospitals between 2014.01 and 2021.12 and divided the development and validation of clinical prediction models into a training cohort (*n* = 3173) and a validation cohort (*n* = 2159). Characteristic variables were selected from the training cohort using the least absolute shrinkage and selection operator (LASSO) algorithm and multivariable logistic regression analysis. A nomogram model was developed, and its performance was evaluated regarding calibration, discrimination, and clinical utility.

**Results:**

We found the best subset of risk factors based on clinical characteristics and laboratory variables, including age, congestive heart failure (CHF), previous AIS/transient ischemia attack (TIA), national institutes of health stroke scale (NIHSS) score, C-reactive protein (CRP), and B-type natriuretic peptide (BNP). A predictive model was developed. The model showed good calibration and discrimination, with calibration values of Hosmer-Lemeshow χ^2^ = 4.813, *P* = 0.732 and Hosmer-Lemeshow χ^2^ = 4.248, *P* = 0.834 in the training and validation cohorts, respectively. The area under the ROC curve (AUC) was 0.815, 95% CI (0.777–0.853) and 0.808, 95% CI (0.770–0.847). The inclusion of neuroimaging variables significantly improved the performance of the integrated model in both the training cohort (AUC. 0.846 (0.811–0.882) vs. 0.815 (0.777–0.853), *P* = 0.001) and the validation cohort (AUC: 0.841 (0.804–0.877) vs. 0.808 (0.770–0.847), *P* = 0.001). The decision curves showed that the integrated model added more net benefit in predicting the incidence of AFDAS.

**Conclusion:**

Predictive models based on clinical characteristics, laboratory variables, and neuroimaging variables showed good calibration and high net clinical benefit, informing clinical decision-making in diagnosing and treating patients with AFDAS.

## Introduction

Atrial fibrillation (AF) is more likely to lead to the occurrence and recurrence of stroke and other adverse events (1). The risk of stroke recurrence in patients with AFDAS is similar to that in patients with AF known before stroke (2). Therefore, early diagnosis of AFDAS and administration of anticoagulation therapy is effective in reducing stroke recurrence rates and mortality (3–5). Currently, up to a quarter of patients have AFDAS (3) and most AFDAS is paroxysmal and asymptomatic. More than 50% of AFDAS last <30 s, which may increase the risk of cryptogenic stroke (CS) (6). The detection rate of AF in patients with CS remains relatively low in large-scale population studies (7). Current guidelines recommend extending the duration of ECG monitoring by 1 week and beyond to improve AF detection (8). Due to limited healthcare resources at all levels, it is impossible to sequentially use all cardiac monitoring techniques for real-time follow-up of patients during hospitalization and after discharge. Therefore, developing a targeted selection strategy is essential for identifying patients at risk for AFDAS, implementing tighter cardiac monitoring, and improving prognosis.

Current research on this topic is continuously updated, many potential markers are summarized (9), and AF risk prediction models are developed. The study by Seo et al. (10) was based on logistic regression to construct a post-stroke AF prediction model. They only evaluated the model by the receiver operator characteristic (ROC) curves without assigning and visualizing risk variables, which was inconvenient for clinicians. Similarly, some scholars combined CHADS_2_ or CHA_2_DS_2_-VAS_C_ score and neuroimaging features to build a prediction model in AIS patients with newly detected AF. Only the C-statistic was used to test the clinical prediction model. The C statistics after the combination were 0.74 and 0.75, respectively (11), with limited predictive performance. In a study to estimate the incidence of AFDAS by electronic medical record algorithms, the AFDAS predictive performance of different scoring systems was validated. Several validation methods were used, including C-index, decision curve analysis (DCA), net reclassification index (NRI), and integrated discrimination improvement (IDI). However, the highest C-index for CHASE-LESS score was 0.741 (12), and the predictive performance was limited by the lack of imaging variables. To date, specific and practical prediction methods are still lacking.

Therefore, developing and validating simple and easy-to-use nomogram models is crucial. The development of prediction models facilitates clinicians in identifying patients at high risk for AFDAS, adjusting cardiac monitoring protocols, and providing information for patient treatment decisions.

## Materials and methods

### Study design and procedure

**Figure 1:**
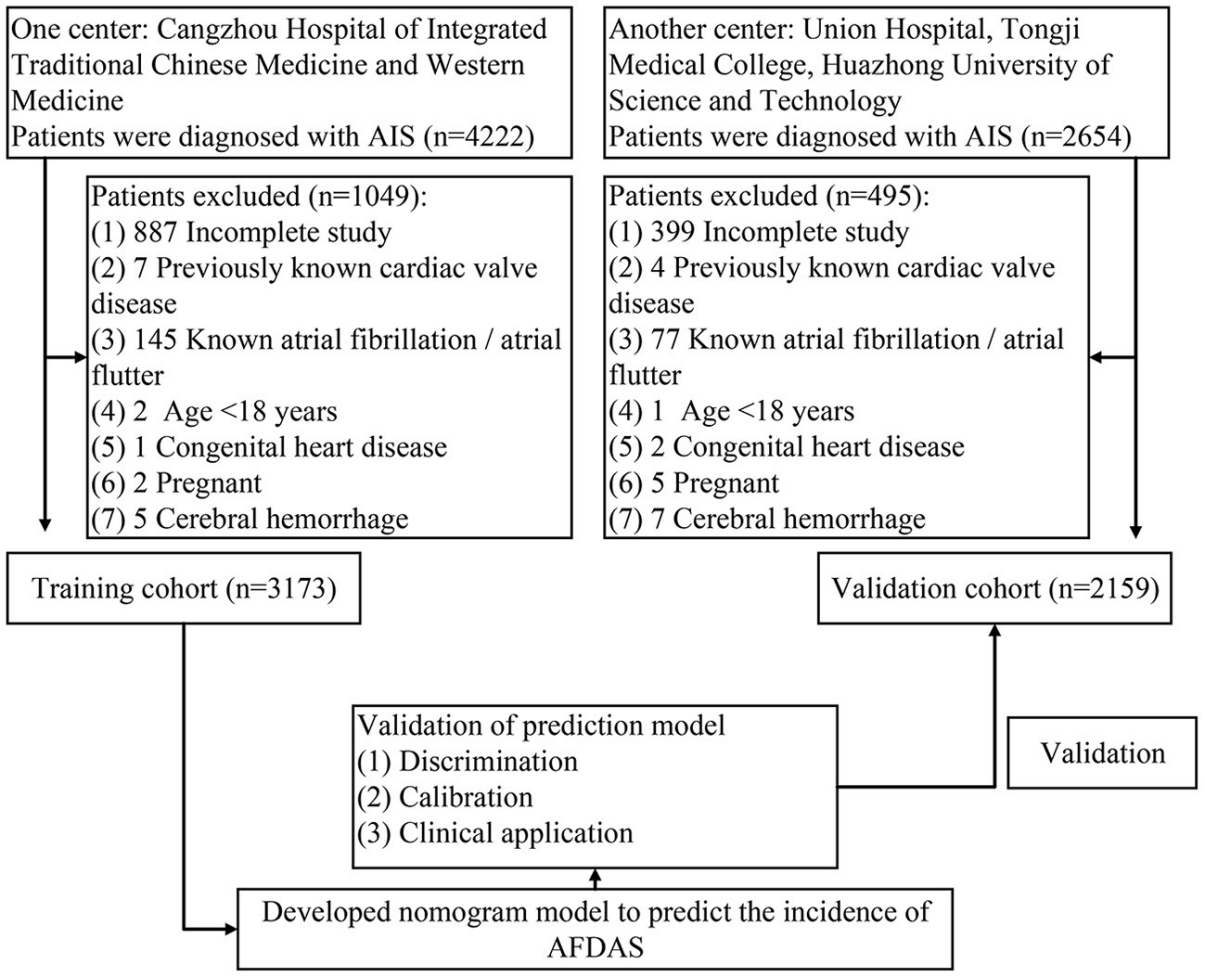
The flow chart of developing and validating the nomogram.

This retrospective study reviewed 4222 patients diagnosed with AIS at Cangzhou Hospital of Integrated Traditional Chinese Medicine and Western Medicine from 2014.01 to 2021.12. Inclusion criteria: (i) patients with AIS need to be diagnosed by specialist neurological and imaging examinations, and they should also have symptoms of acute focal neurological dysfunction lasting more than 24 h (13–15); (ii) ECG monitoring by three modalities: conventional ECG, ambulatory ECG, and continuous cardiac telemetry after admission. In order not to affect the modeling and outcome, some patients were ruled out: (i) previous AF or atrial flutter; (ii) heart valve disease; (iii) congenital heart disease; (iv) cases with incomplete studies; (v) cerebral hemorrhage; (vi) pregnancy; (vii) patients under 18 years of age. Ultimately, 3173 patients entered the training cohort. In the same way, 2159 patients were selected for inclusion in the validation cohort in the Union Hospital of Tongji Medical College, Huazhong University of Science and Technology. All patients were followed up by telephone at 28-day and 90-day after stroke. The flow chart of the study was shown in Figure 1. The study protocol was approved by the ethics committee of Cangzhou Hospital of Integrated Traditional Chinese Medicine and Western Medicine, and written informed consent was waived (No. 2022-0197-01).

We retrieved information on patients with AIS, including essential admission characteristics such as age, sex, history, NIHSS score, 12-lead body surface ECG, continuous cardiac telemetry/bedside ECG monitoring, ambulatory ECG, brain CT and MRI scans (including T1-weighted imaging, T2-weighted imaging, fluid-attenuated inversion recovery imaging, diffusion-weighted imaging (DWI) and apparent diffusion coefficient sequences), and blood analysis. AFDAS was determined based on 12-lead ECG and ambulatory ECG reports and nursing records from the case data, and the results were diagnosed by neurologists and cardiologists. AFDAS was defined as AF that did not occur before admission and was only newly detected after AIS during admission, including persistent episodes of AF>30 seconds (16). Non-AFDAS, on the other hand, was no AF detected before and during admission. All laboratory data were selected for results within 24 h of admission. If multiple values were reported for a variable within 24 h of admission, the worst one was selected for analysis.

Neuroimaging (CT or MRI) presentations were evaluated by specialized imaging physicians. Acute lesions appear as cerebral hypodensity with cytotoxic edema on CT. Acute lesions on MRI are identified by restricted diffusion on DWI sequences and low signal on apparent diffusion coefficient sequences. Chronic cerebral infarction is defined on brain CT as a hypointense lesion without associated cytotoxic edema type. MRI of chronic cerebral infarction shows inversion recovery of fluid attenuation or increased signal on T2-weighted sequences but no signs of diffusion restriction on DWI sequences (11, 15). We documented lesion location (cortical infarction, subcortical infarction, brainstem infarction, cerebellar infarction) and multiple lesions (≥1 primary lesion [left and right carotid arteries and vertebrobasilar artery] vessel regions) (11).

### Outcomes

The primary outcome was the incidence of AFDAS during hospitalization. Secondary outcomes were days of hospitalization, 28-day and 90-day mortality, and in-hospital mortality.

### Predictors acquisition and development of prediction models

We used the LASSO and multivariable logistic regression analysis to screen the training cohort for relevant factors and obtain the seven risk factors. We developed a prediction model based on clinical characteristics and laboratory variables, Model 1. A new prediction model based on Model 1 was developed in conjunction with neuroimaging variables, Model 2. To evaluate the performance of both models, we performed calibration, discrimination, and clinical utility.

### Statistical analysis

Statistical analysis was conducted with SPSS (IBM SPSS Statistics 26.0, SPSS Inc., Chicago, IL) and R language (version 4.1.3, www.R-project.org/). The R packages used in our study were displayed in Supplementary Table 1. In univariate analysis with and without AFDAS, continuous variables conformed to a normal distribution were subjected to Student's *t*-test, expressed as mean ± standard deviation. Continuous variables that did not conform to a normal distribution were subjected to the non-parametric test (Wilcoxon rank sum test), described as median and interquartile (25th to 75th percentile) range (IQR). Categorical variables were subjected to the Chi-square test or Fisher's exact test, expressed as frequencies and percentages. All statistical tests were two-sided, and statistical significance was set at 0.05. The study data from Cangzhou Hospital of Integrated Traditional Chinese Medicine and Western Medicine was used as the training cohort and the dataset from Wuhan Union Hospital was used as the validation cohort. Characteristic variables were selected from the training cohort using the LASSO algorithm and multivariable logistic regression analysis.

To build the predictive model for AFDAS, we weighted the coefficients of the variables screened out of the training cohort above to construct the nomogram model for AFDAS. The predictive performance of the model is assessed by means of ROC curves, which compare the area under the curve. In addition, validation of the model is performed in the validation cohort. The calibration curve is achieved by depicting a smooth non-parametric calibration curve and a fitted logistic calibration curve for the validation cohort. For the assessment of clinical utility, decision curve analysis is one of the most appropriate assessment methods. By quantifying the clinical utility of the model, decision curves can show the net benefit and risk threshold probabilities. In addition, we used the median and interquartile (25th−75th percentile) range (IQR) and chi-square tests to analyse outcome variables such as length of stay in hospital and in-hospital mortality in stroke patients.

## Results

### Clinical characteristics

A total of 5332 patients were eligible for this study, of which 3480 (65.3%) were male and 1852 (34.7%) were female, with a mean age of 62.93 ± 12.55 years. Among these patients, 384 (7.2%) developed AFDAS during hospitalization. All clinical variables were not significantly different between the training cohort (*n* = 3173) and the validation cohort (*n* = 2159) (*P* > 0.05). The incidence of AFDAS was similar in both cohorts, with 225 (7.1%) in the training cohort and 159 (7.4%) in the validation cohort (Supplementary Table 2). In the training cohort, 2948 (92.9%) had non-AFDAS and 225 (7.1%) had AFDAS. Baseline characteristics were shown in Table 1. Among the different subtypes of TOAST typology, 317 (5.95%) patients with large-artery atherosclerosis had AF, of which 313 (81.5%) had received anticoagulation. Undetermined etiology had 14 (0.26%) patients with AF, and all had received anticoagulation (Supplementary Table 3).

**Table 1:** Baseline characteristics of patients with non-AFDAS and AFDAS in training cohort.

**Variables**	**All patients (*n* = 3173)**	**non-AFDAS (*n* = 2948)**	**AFDAS (*n* = 225)**	***P***
Gender, *n* (%)				0.688
Male^§^	2048 (64.5)	1900 (64.5)	148 (65.8)	
Female^§^	1125 (35.5)	1048 (35.5)	77 (34.2)	
Age (years)^†^	63.14 ± 12.83	62.64 ± 12.72	69.69 ± 12.46	< 0.001
Physiological data on admission				
Heart rates (beats/min)^†^	104.87 ± 10.10	104.85 ± 10.10	105.20 ± 10.16	0.617
MAP (mm Hg)^†^	94.44 ± 6.08	94.41 ± 5.92	94.76 ± 8.01	0.409
BMI (kg/m^2^)^†^	21.08 ± 1.95	21.07 ± 1.95	21.24 ± 2.01	0.211
Comorbidity, *n* (%)				
Hypertension^§^	560 (17.6)	507 (17.2)	53 (23.6)	0.016
Coronary artery disease^§^	192 (6.1)	174 (5.9)	18 (8.0)	0.203
CHF^§^	258 (8.1)	217 (7.4)	41 (18.2)	< 0.001
Diabetes mellitus^§^	442 (13.9)	405 (13.7)	37 (16.4)	0.258
COPD^§^	279 (8.8)	258 (8.8)	21 (9.3)	0.767
Hyperlipidemia^§^	692 (21.8)	631 (21.4)	61 (27.1)	0.046
Previous AIS/TIA^§^	421 (13.3)	372 (12.6)	49 (21.8)	< 0.001
Hepatic insufficiency^§^	250 (7.9)	235 (8.0)	15 (6.7)	0.484
Renal insufficiency^§^	331 (10.4)	310 (10.5)	21 (9.3)	0.576
Neuroimaging, *n* (%)				
Cortical infarction^§^	1031 (32.5)	905 (30.7)	126 (56.0)	< 0.001
Subcortical infarction^§^	1680 (52.9)	1563 (53.0)	117 (52.0)	0.768
Brainstem infarction^§^	542 (17.1)	507 (17.2)	35 (15.6)	0.528
Cerebellar infarction^§^	401 (12.6)	366 (12.4)	35 (15.6)	0.172
Multiple lesions of arterial territory^§^	986 (31.1)	905 (30.7)	81 (36.0)	0.098
Stroke location, *n* (%)				0.258
Left-sided^§^	1682 (53.0)	1558 (52.8)	124 (55.1)	
Right-sided^§^	1213 (38.2)	1125 (38.2)	88 (39.1)	
Bilateral^§^	278 (8.8)	265 (9.0)	13 (5.8)	
Subtype of stroke, *n* (%)				0.381
Large-artery atherosclerosis^§^	2492 (78.5)	2306 (78.2)	186 (82.7)	
Cardioembolism^§^	279 (8.8)	266 (9.0)	13 (5.8)	
Small-artery occlusion^§^	134 (4.2)	123 (4.2)	11 (4.9)	
Other determined etiology^§^	124 (3.9)	117 (4.0)	7 (3.1)	
Undetermined etiology^§^	144 (4.5)	136 (4.6)	8 (3.6)	
Severity on admission				
NIHSS score*	8.00 (5.00, 10.00)	8.00 (5.00, 10.00)	10.00 (8.00, 16.00)	< 0.001
GCS score*	9.00 (7.00, 11.00)	9.00 (7.00, 11.00)	10.00 (7.00, 11.00)	0.190
Laboratory tests				
White blood cell count ( × 10^9^/L)*	6.74 (5.80, 8.55)	6.74 (5.74, 8.55)	7.10 (6.06, 8.64)	0.180
Hemoglobin (g/L)*	114.00 (110.00, 117.00)	114.00 (110.00, 117.00)	113.00 (110.00, 116.50)	0.175
Platelet count ( × 10^9^/L)*	156.00 (99.00, 165.00)	156.00 (99.00, 165.00)	156.00 (98.00, 164.00)	0.881
Red blood cell ( × 10^12^/L)*	4.05 (3.47, 4.49)	4.05 (3.49, 4.48)	4.03 (3.25, 4.59)	0.790
Serum creatinine (μmol/L)*	80.44 (72.91, 86.72)	80.28 (72.82, 86.72)	81.73 (74.12, 86.81)	0.161
Blood urea nitrogen (mmol/L)*	5.70 (4.30, 6.90)	5.70 (4.30, 6.80)	6.00 (4.50, 8.05)	0.047
ALT (U/L)*	35.00 (24.00, 46.00)	35.00 (24.00, 46.00)	32.00 (16.00, 43.00)	< 0.001
Bilirubin (μmol/L)*	11.70 (8.30, 16.50)	11.70 (8.20, 16.60)	11.50 (8.35, 15.74)	0.453
Albumin (g/L)*	40.50 (37.30, 41.60)	40.50 (37.30, 41.60)	40.50 (37.40, 42.15)	0.776
Cardiac troponin I (ng/mL)*	0.010 (0.001, 0.149)	0.010 (0.001, 0.150)	0.012 (0.001, 0.135)	0.260
Creatine kinase (U/L)*	65.00 (42.00, 104.00)	65.00 (42.00, 102.75)	74.00 (45.00, 127.00)	0.014
Triglyceride (mmol/L)*	1.28 (0.93, 1.82)	1.28 (0.93, 1.81)	1.36 (0.91, 2.07)	0.103
Total cholesterol (mmol/L)*	3.93 (3.25, 4.75)	3.92 (3.25, 4.72)	4.02 (3.24, 5.05)	0.117
HDL-C (mmol/L)*	1.10 (0.90, 1.30)	1.10 (0.90, 1.30)	1.10 (0.90, 1.40)	0.343
LDL-C (mmol/L)*	2.18 (1.69, 2.78)	2.18 (1.70, 2.76)	2.22 (1.53, 2.97)	0.766
BNP (pg/mL)*	94.89 (80.46, 110.05)	94.60 (80.94, 108.49)	119.60 (66.25, 238.70)	< 0.001
Fibrinogen (g/L)*	3.65 (2.94, 4.71)	3.64 (2.94, 4.72)	3.70 (2.98, 4.69)	0.479
APTT (s)*	36.80 (33.80, 40.70)	36.70 (33.80, 40.70)	38.3 (34.70, 42.55)	0.001
PT (s)*	13.50 (12.90, 14.70)	13.60 (12.90, 14.78)	13.40 (12.70, 14.70)	0.172
INR*	1.05 (0.98, 1.17)	1.05 (0.98, 1.17)	1.05 (0.98, 1.23)	0.761
D-dimer (mg/L)*	1.14 (0.45, 2.69)	1.09 (0.45, 2.61)	1.51 (0.57, 4.74)	< 0.001
Lactic acid(mmol/L)*	4.40 (3.70, 5.10)	4.40 (3.70, 5.10)	4.40 (3.70, 5.10)	0.682
Procalcitonin (μg/L)*	0.17 (0.13, 0.77)	0.17 (0.13, 0.77)	0.17 (0.13, 0.79)	0.596
CRP (mg/L)*	21.80 (4.09, 58.60)	18.10 (3.65, 53.43)	49.50 (23.20, 92.50)	< 0.001

^†^Normally distributed continuous variables are presented as means with standard deviations and analyzed by Student's *t*-test.

^*^Non-normally distributed continuous variables are presented as medians with interquartile ranges and analyzed by non-parametric test.

^§^Categorical variables are presented as frequencies with percentages and analyzed by Chi-square test or Fisher' s exact test.

AFDAS, Atrial Fibrillation Detected After Stroke; MAP, mean arterial pressure; BMI, body mass index; CHF, Congestive heart failure; COPD, chronic obstructive pulmonary disease; AIS, acute ischemic stroke; TIA, transient ischemic attack; NIHSS, national institute of health stroke scale; GCS, glasgow coma scale; ALT, alanine aminotransferase; HDL-C, high-density lipoprotein cholesterol; LDL-C, low-density lipoprotein cholesterol; BNP, B-type natriuretic peptide; APTT, activeated partial thromboplasting time; PT, prothrombin time; INR, international normalized ratio; CRP, C-reaction protein.

### Feature selection of independent predictors

**Figure 2:**
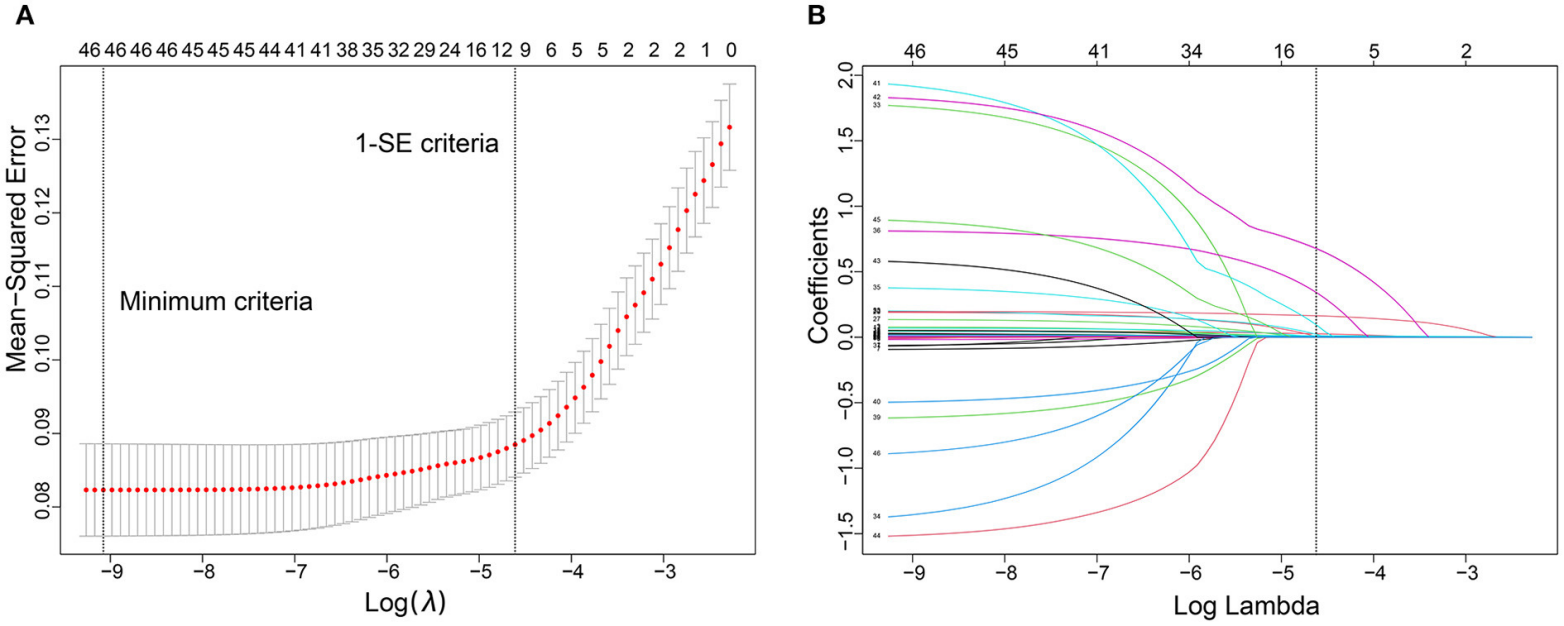
Predictor selection using the least absolute shrinkage and selection operator (LASSO) logistic regression model. **(A)** Identification of the optimal penalization coefficient lambda (λ) in the LASSO model. The dotted vertical line was plotted at the value selected using 10-fold cross-validation, for which the optimal λ resulted in 10 non-zero coefficients. **(B)** LASSO coefficient profiles of the 45 predictors. A coefficient profile plot was produced against the log (λ) sequence.

**Figure 3:**
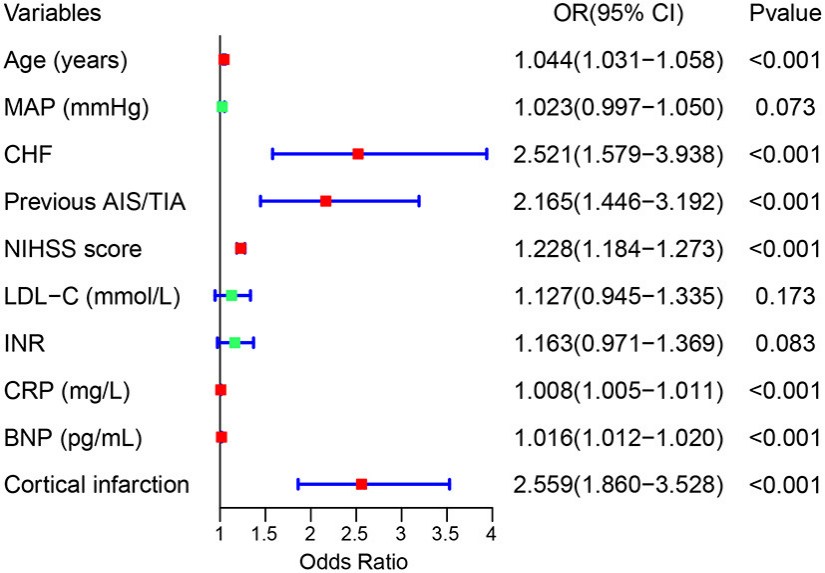
Forest plot of risk predictors in AFDAS with AIS.

We used the LASSO regression model to build predictor classifiers in the training cohort. Finally, 10 non-zero characteristic variables were selected among 45 variables (Figures 2A,B), including age, mean artery pressure (MAP), CHF, previous AIS/TIA, cortical infarction, NIHSS score, low-density lipoprotein cholesterol (LDL-C), international normalized ratio (INR), CRP, and BNP (Table 2). To screen out the strongest predictors and construct a clinical prediction model, we used a multivariable logistic regression analysis (forest plot). The analysis revealed that age (1.044 [1.031–1.058]; *P* < 0.001), CHF (2.521 [1.579–3.938]; *P* < 0.001), previous AIS/TIA (2.165 [1.446–3.192]; *P* < 0.001), NIHSS score (1.228 [1.184–1.273]; *P* < 0.001), CRP (1.008 [1.005–1.011]; *P* < 0.001), BNP (1.016 [1.012–1.020]; *P* < 0.001) and cortical infarction (2.559 [1.860–3.528]; *P* < 0.001) were significantly associated with AFDAS in the training cohort (Figure 3).

**Table 2:** LASSO regression coefficients and lambda.1-SE values in the training cohort.

**Variables**	**Coefficients**	**Lambda.1-SE**	**log (Lambda)**
Age (years)	0.026	0.010	– 4.611
Physiological data on admission			
MAP (mm Hg)	0.003		
Comorbidity			
CHF	0.337		
Previous AIS/TIA	0.089		
Neuroimaging			
Cortical infarction	0.674		
Severity on admission			
NIHSS score	0.164		
Laboratory tests			
LDL-C (mmol/L)	0.002		
INR	0.020		
CRP (mg/L)	0.005		
BNP (pg/mL)	0.011		

MAP, mean arterial pressure; CHF,Congestive heart failure; AIS, acute ischemic stroke; TIA, transient ischemic attack; NIHSS, national institute of health stroke scale; LDL-C, low-density lipoprotein cholesterol; INR, international normalized ratio; CRP, C-reaction protein; BNP, B-type natriuretic peptide.

### Prediction model based on simplified clinical characteristics and laboratory variables

**Figure 4:**
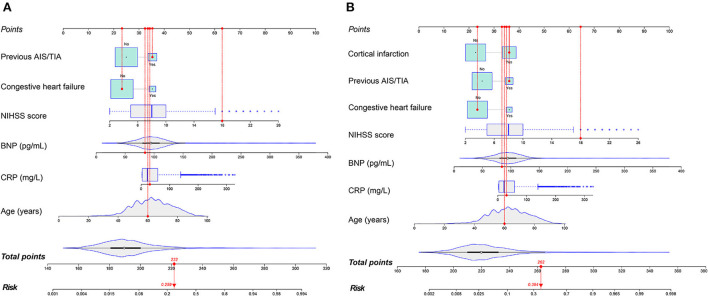
The nomograms to predict the probability of AFDAS after AIS patients from the training cohort. The patient was 60 years old with a history of stroke and no history of heart failure. During hospitalization the NIHSS score was 18, the C-reactive protein was 30.4 mg/L and the BNP was 84.3 pg/mL. The patient had a total score of 222 and a probability of atrial fibrillation of 25.9% (model 1) **(A)**. When the neuroimaging variable (Cortical infarction) was added, the patient had a total score of 262 and a 38.4% probability of atrial fibrillation (model 2) **(B)**.

The prediction model consisted of six predictors, including age, CHF, previous AIS/TIA, NIHSS score, CRP and BNP, through the screening of variables by LASSO and multivariable logistic regression analysis described above (Table 3). We obtained regression coefficients for the variables by multivariable logistic regression analysis and proposed a risk score formula: risk score = – 9.139 + 0.046 (age) + 0.902 (if congestive heart failure was positive) + 0.774 (if previous AIS/TIA was true) + 0.208 (NIHSS score) + 0.008 (CRP) + 0.017 (BNP). Predicted risk =1/(1 + e^−*riskscore*^). The model containing the above predictors was developed and presented as a nomogram (Figure 4A).

**Table 3:** Risk factors and development of predictive models for AFDAS in training cohort.

**Variables**	**Model 1**			**Model 2**		
	**β**	**OR(95% CI)**	***P***	**β**	**OR(95% CI)**	***P***
Intercept	– 9.139		< 0.001	– 8.428		< 0.001
Age (years)	0.046	1.045(1.032–1.058)	< 0.001	0.043	1.044(1.031–1.058)	< 0.001
CHF	0.902	2.465(1.582–3.842)	< 0.001	0.915	2.498(1.453–3.822)	< 0.001
Previous AIS/TIA	0.774	2.168(1.463–3.164)	< 0.001	0.778	2.179(1.460–3.204)	< 0.001
NIHSS score	0.208	1.232(1.189–1.276)	< 0.001	0.207	1.230(1.187–1.275)	< 0.001
CRP (mg/L)	0.008	1.008(1.006–1.011)	< 0.001	0.008	1.008(1.006–1.011)	< 0.001
BNP (pg/mL)	0.017	1.017(1.014–1.021)	< 0.001	0.016	1.016(1.013–1.020)	< 0.001
Cortical infarction	NA	NA	NA	0.966	2.628(1.914–3.617)	< 0.001

model 1: based on simplified Clinical features and laboratory variables alone.

model 2: based on simplified Clinical features, laboratory variables and neuroimaging variables.

OR, odds ratio; CHF, Congestive heart failure; AIS, acute ischemic stroke; TIA, transient ischemic attack; NIHSS, national institute of health stroke scale; CRP, C-reaction protein; BNP, B-type natriuretic peptide.

### Validation of prediction models

**Figure 5:**
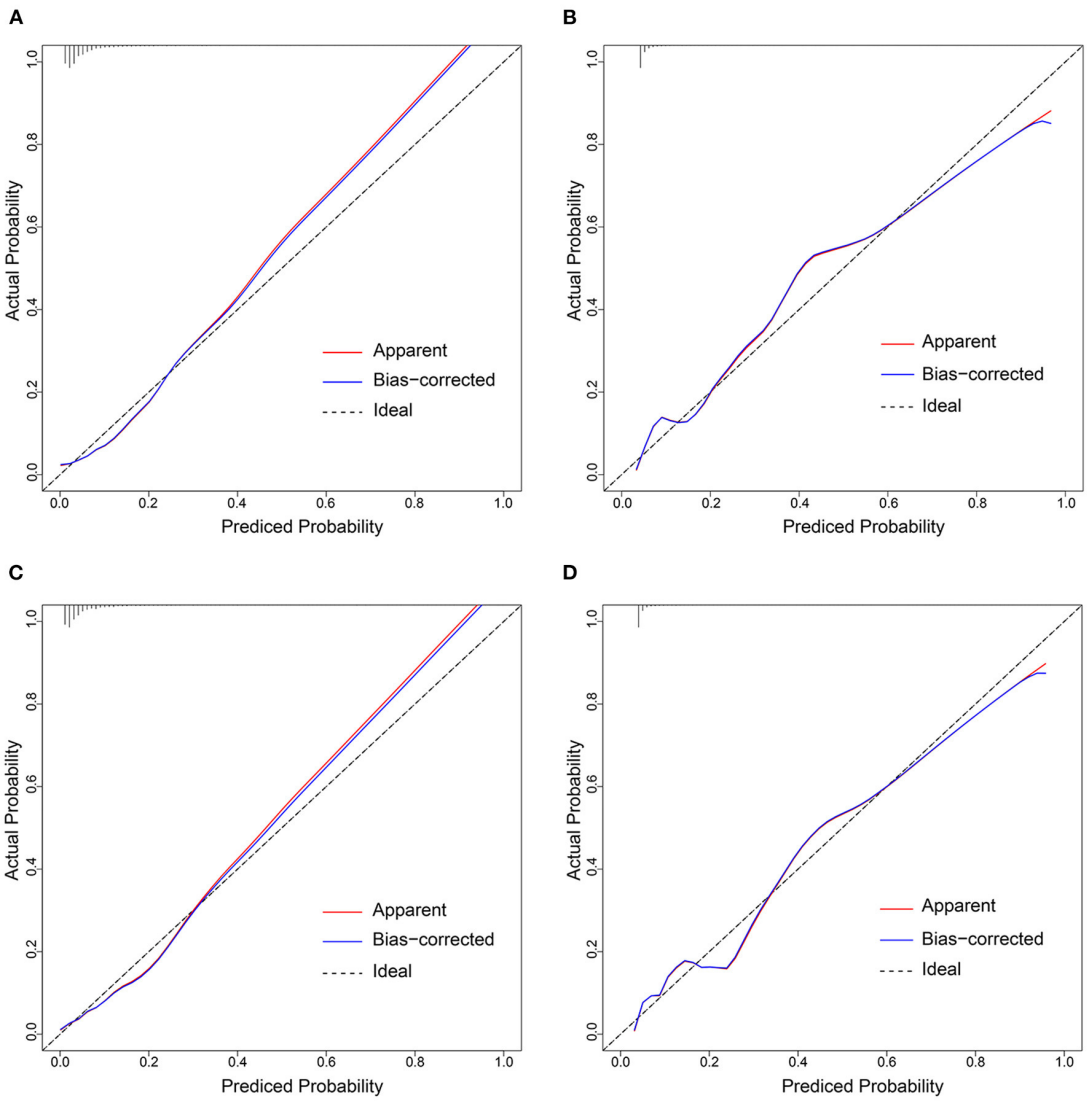
Calibration curves of the prediction models in each cohort. Calibration curves depict the calibration of prediction models in terms of the agreement between the predicted risks of AFDAS and observed outcomes of AFDAS. The x-axis represents the predicted AFDAS risk and the y-axis represents the actual AFDAS rate. The diagonal dotted line represents a perfect prediction by an ideal model. The red solid line represents the performance of our prediction models. A closer fit to the diagonal dotted line represents a better prediction. **(A,B)** represents the calibration curve of Model 1 in the training cohort and validation cohort; **(C,D)** represents the calibration curve of Model 2 in the training cohort and validation cohort.

**Figure 6:**
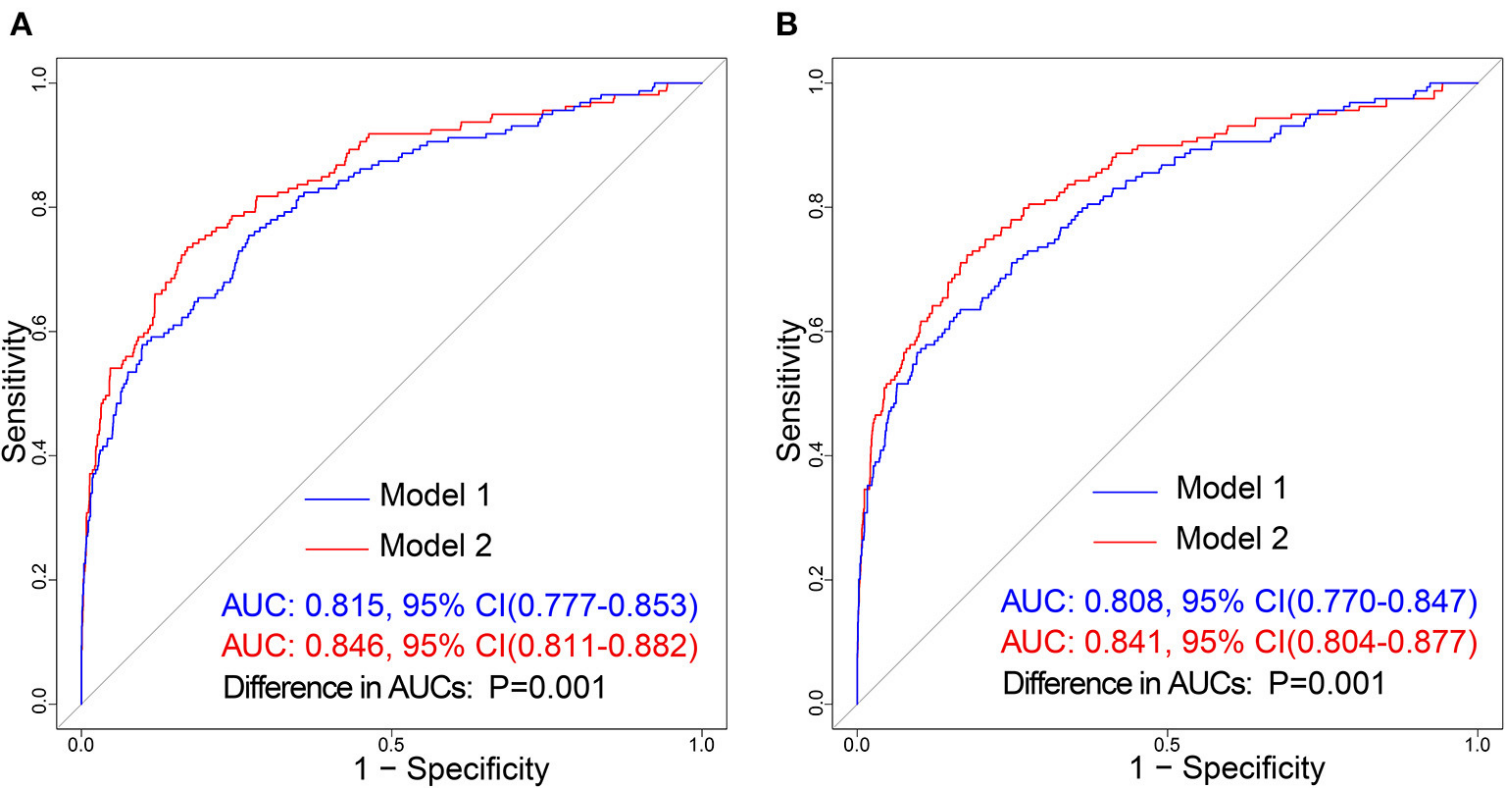
Receiver operating characteristic (ROC) curve of the prediction models in each cohort. The blue line and red line represent Model 1 and Model 2, respectively. **(A)** Represents ROC curve of our prediction models in the training cohort; **(B)** Represents the ROC curve of models in the validation cohort.

In the training cohort, there was a well-calibrated between prediction and observation (Hosmer-Lemeshow χ^2^ = 4.813, *P*= 0.732) (Figure 5A). The AUC for the training cohort was 0.815, 95% CI (0.777–0.853) (Figure 6A). The validation cohort also showed a good calibration (Hosmer-Lemeshow χ^2^ = 4.248, *P* = 0.834) (Figure 5B). The AUC was 0.808, 95% CI (0.770–0.847) (Figure 6B).

### Incremental prediction of the above model by neuroimaging variables

Imaging variables were analyzed by imaging physicians in stroke patients and Cortical infarction was significantly associated with the development of atrial fibrillation after univariate group comparisons and multivariable logistic regression analysis. To evaluate the incremental predictive value of neuroimaging variables, the AFDAS prediction model was developed using neuroimaging variables combined with Model 1, i.e., Model 2. Finally, 7 optimal predictors were selected, including age, CHF, previous AIS/TIA, cortical infarction, NIHSS score, CRP, and BNP (Table 3). The risk score equation for Model 2 was: risk score = – 8.428 + 0.043 (age) + 0.915 (if congestive heart failure was positive) + 0.778 (if previous AIS/TIA was true) + 0.207 (NIHSS score) + 0.008 (CRP) + 0.016 (BNP) + 0.966 (if cortical infarction was positive). Predicted risk = 1/(1 + e^−*riskscore*^). A model combining the above predictors was developed and presented as a nomogram (Figure 4B).

The training cohort (Hosmer-Lemeshow χ^2^ = 4.956, *P* = 0.713) and the validation cohort (Hosmer-Lemeshow χ^2^ = 4.311, *P* = 0.828) showed well-calibrated between prediction and observation (Figures 5C,D). After the addition of cortical infarction variables, Model 2 showed significantly higher discrimination between the training cohort (AUC: 0.846, 95% CI (0.811–0.882) vs. 0.815, 95% CI (0.777–0.853), *P* = 0.001) (Figure 6A) and the validation cohort (AUC: 0.841, 95% CI (0.804–0.877) vs. 0.808, 95% CI (0.770–0.847), *P* = 0.001) (Figure 6B, Supplementary Table 4).

### Clinical utility and improvement capacity of the model

**Figure 7:**
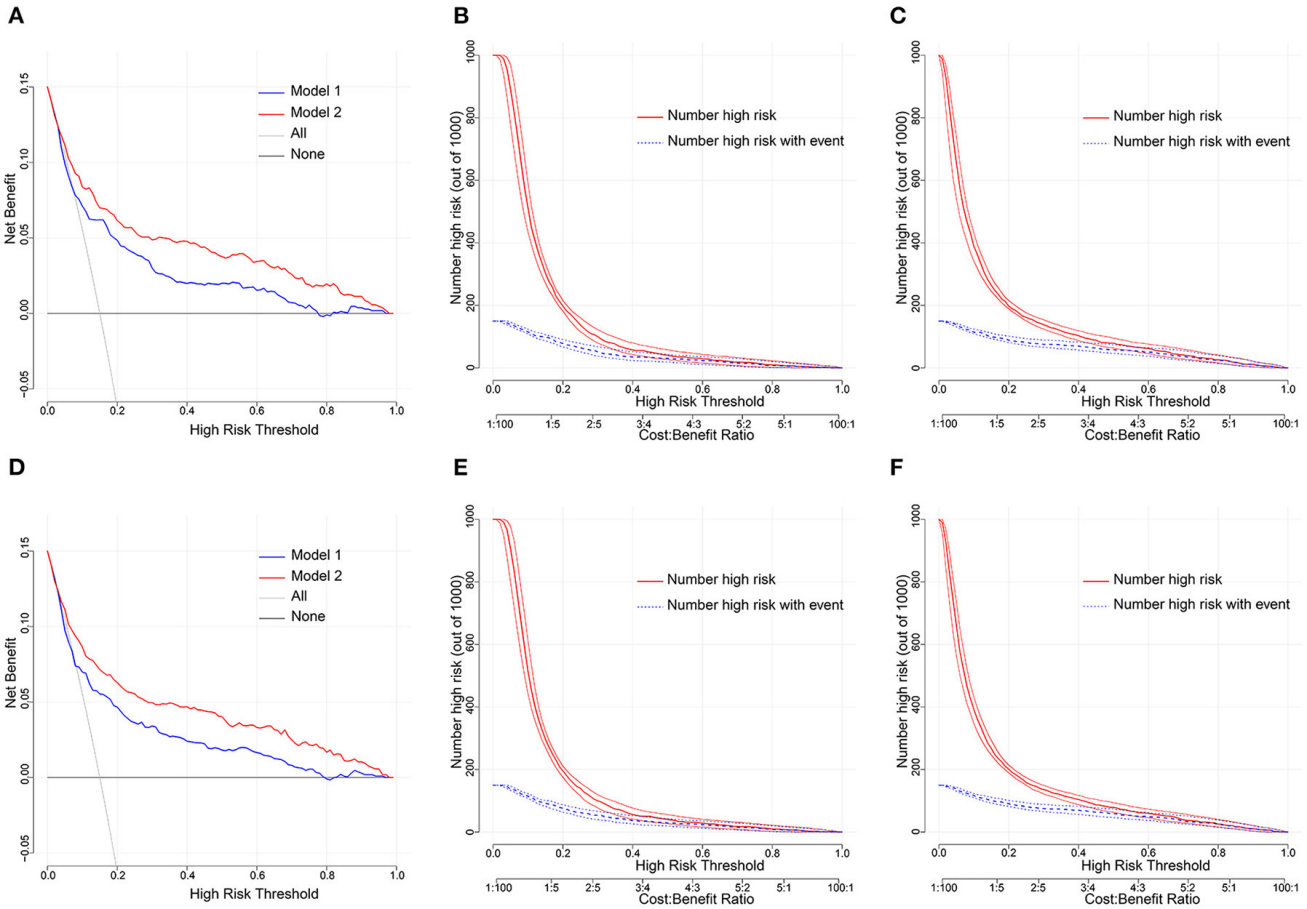
**(A,D)** Decision curve analysis for the Model 1 and Model 2 nomogram in the training and validation cohort. The y and x axis means the true and false positive rate of the risk prediction of AFDAS patients, respectively. The blue line represents the Model 1 nomogram. The red line represents the Model 2 nomogram. **(B,C)** Clinical impact curve in the training cohort; **(E,F)** Clinical impact curve in the validation cohort.

**Figure 8:**
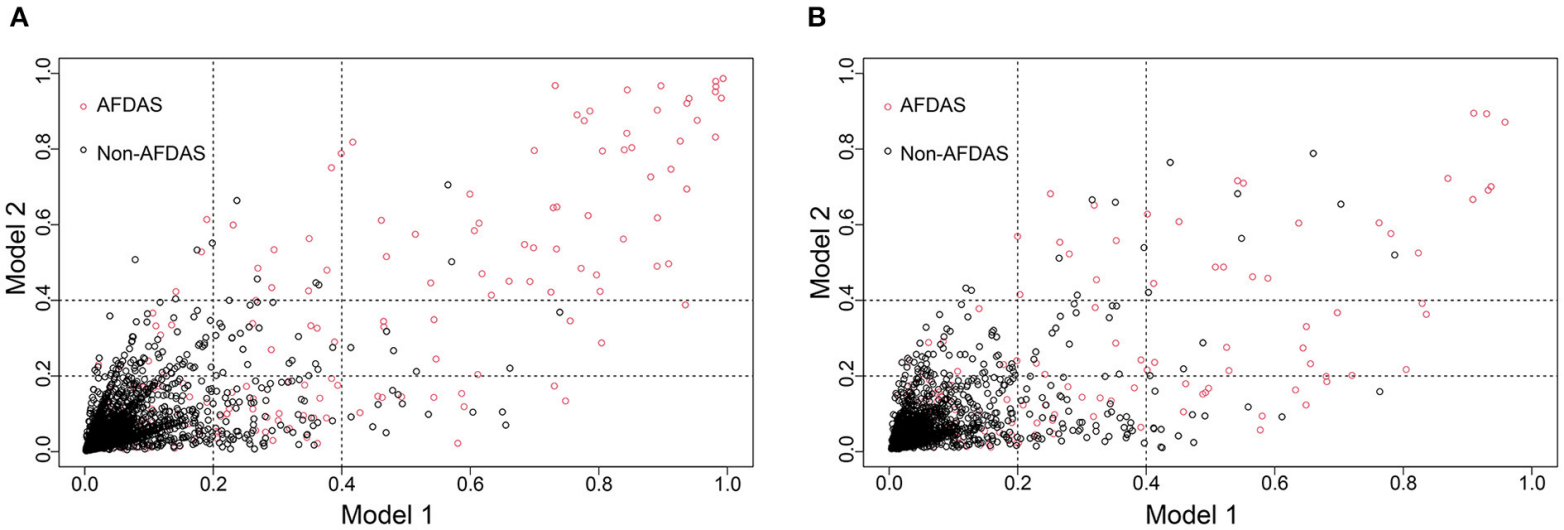
Model 1 and Model 2 comparison based on NRI in training cohort **(A)** and validation cohort **(B)**. Model 2 is 0.105 better than Model 1 in training cohort. Model 2 is 0.086 better than Model 1 in validation cohort.

DCA is one of the methods to assess the clinical utility of prediction models. More significant net clinical benefit was obtained for model 2 than model 1 in the training cohort. Similar results were observed in the validation cohort (Figures 7A,D). The clinical impact curves also showed that the number of patients predicted to develop AF converged with those who developed AF within this risk threshold, indicating the model's significant predictive power and good clinical utility (Figures 7B,C,E,F). To compare the improvement ability of the two models, NRI and IDI were used. Model 2 was compared with Model 1, with NRI of 0.105 (0.025–0.185) in the training cohort and 0.086 (0.026–0.147) in the validation cohort. IDI was 0.106 (0.074–0.139) in the training cohort and 0.031 (0.016–0.047) in the validation cohort (Figures 8A,B and Table 4). Therefore, Model 2 had better predictive performance for AFDAS incidence compared with Model 1.

**Table 4:** Comparison of the prediction ability between Model 1 and Model 2 through NRI and IDI.

**Variables**	**Value**	**95% CI**	***P***
Training Cohort			
NRI	0.105	0.025–0.185	0.010
IDI	0.106	0.074–0.139	< 0.001
Validation Cohort			
NRI	0.086	0.026–0.147	0.005
IDI	0.031	0.016–0.047	< 0.001

NRI, net reclassification index; IDI, integrated discrimination index.

### Outcomes

**Figure 9:**
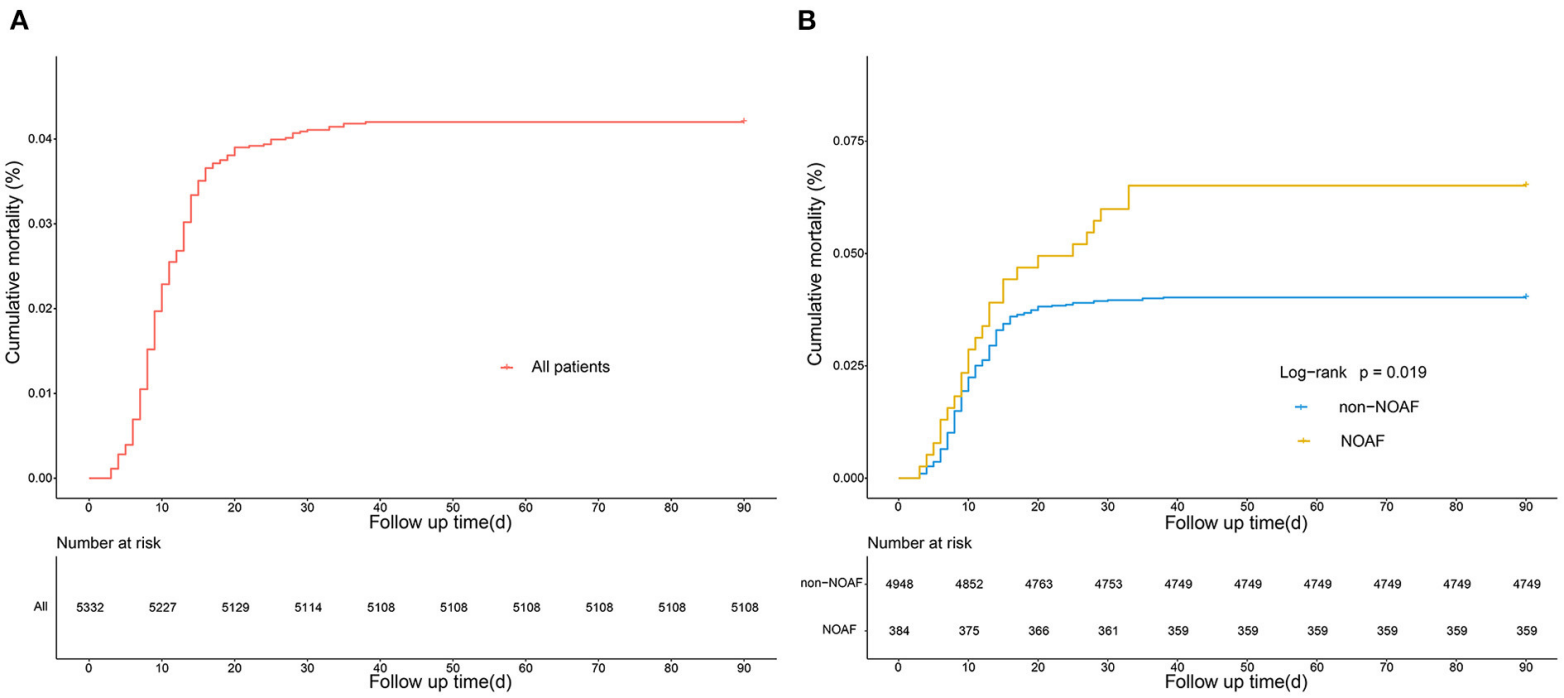
Kaplan-Meier curves showing the cumulative mortality of all the patients of AFDAS **(A)**, and the comparison of cumulative mortality in patients with and without AFDAS **(B)**.

Of the 5332 patients, 384 (7.2%) developed AFDAS after AIS. By univariate analysis, patients with AFDAS had a poor prognosis with 11 (8–18) days of hospitalization, significantly higher than the non-AFDAS group. One hundred (1.9%) patients died during hospitalization in the study population, including 15 (3.9%) in the AFDAS group. In addition, both 28-day and 90-day mortality were statistically significantly higher in the AFDAS group than in the non-AFDAS group (Table 5). The study population's 90-day mortality rate was 4.2%, and the mortality rate increased significantly early in the onset (Figure 9A). Furthermore, the AFDAS group had a significant increase in early mortality rate and a higher 90-day mortality rate than the non-AFDAS group (Figure 9B).

**Table 5:** Outcomes in patients with and without AFDAS.

**Outcome**	**All patients (*n* = 5332)**	**non-AFDAS (*n* = 4948)**	**AFDAS (*n* = 384)**	**χ^2^/Z**	***P***
Hospital stay (days)	11.00 (8.00, 15.00)	11.00(8.00, 14.00)	11.00 (8.00, 18.00)	3.763	< 0.001
28-day mortality, *n* (%)	151 (2.8)	132(2.7)	19 (4.9)	6.733	0.009
90-day mortality, *n* (%)	224 (4.2)	199 (4.0)	25 (6.5)	5.484	0.019
In-hospital mortality, *n* (%)	100 (1.9)	85 (1.7)	15 (3.9)	9.273	0.002

## Discussion

In this study, age, BNP, CRP, CHF, NIHSS score, previous AIS/TIA, and cortical infarction were independent risk factors for AFDAS. We developed and validated a nomogram model for the incidence of AFDAS after AIS based on the above risk factors. After validation, the prediction model showed good calibration, discrimination, and clinical utility.

AF is one of the major risk factors for stroke. Left atrial remodeling characterized by endothelial dysfunction during the first 24 h of an AF episode predisposes to thrombosis (17), which in turn increased the incidence of stroke. One study noted that the risk of stroke was 5 times higher in patients with AFDAS than in those without AFDAS (18), and oral anticoagulation reduced the risk of recurrence in patients with IS who were first diagnosed with AFDAS (19). However, up to 95% of episodes in patients with AFDAS are paroxysmal and asymptomatic (9). Patients with AFDAS are more difficult to be diagnosed and be treated promptly. There is still no consensus on screening strategies and diagnosis of AFDAS, and its prevalence remains uncertain. In our study, the diagnosis rate of AFDAS during hospitalization was 7.2%, similar to previous reports (20, 21). Among the different subtypes of TOAST typology, 317 (5.95%) patients with large-artery atherosclerosis had AF, of which 313 (81.5%) patients with AF were treated with anticoagulation. In addition, some of these patients failed to receive anticoagulation because of the cerebral hemorrhage event that occurred after the stroke. A systematic review and meta-analysis summarized cardiac monitoring methods chronologically into four phases. The detection rate of AFDAS could be improved by extended monitoring mean in phases III and IV, including ambulatory Holter, mobile cardiac outpatient telemetry, external loop recording, and implantable loop recording (21). However, the high cost may not achieve a satisfactory diagnostic yield, depending mainly on appropriate patient selection (22). Therefore, many studies are addressing this area. There is methodological diversity (1, 11, 12, 15, 22–26), different study sample sizes (6, 10, 27), differences in the analysis of multiple factors in the development of AF (28–31), and heterogeneity in its pathophysiological mechanisms (2, 9, 32) in the study process. These caused differences in the findings of AFDAS. Predictive models for AFDAS have been investigated, but there are problems with missing neuroimaging variables, failure to visualize the model and limited predictive power (10–12). In this context, we successfully constructed a new model to predict the occurrence of AFDAS and provide clinical decisions for the treatment of stroke patients. The models could be applied to patients' risk stratification. Patients with a positive prediction of AFDAS may require more sophisticated cardiac exams in the hospital setting.

The occurrence of AFDAS is associated with complex pathophysiological mechanisms, such as neurogenic mechanisms. Autonomic regulation of cardiac rhythm is influenced by the cerebral cortex, and cortical infarction leads to dysregulation of autonomic regulation, which triggers the development of AFDAS (9). In a study of unexplained AFDAS in patients with IS, any imaging manifestation of cortical infarction was associated with AFDAS as an independent predictor (10, 11, 15). In the univariate regression analysis of this study, the number of AFDAS patients with cortical involvement was significantly higher than that of non-AFDAS patients. This result suggested that neurogenic mechanisms may play a role in some AFDAS patients. Likewise, the IS-induced inflammatory response leads to intrinsic autonomic nervous system dysfunction through inflammatory mediator stimulation, causing partial discharges in the cardiac ganglion plexus and reduced heart rate variability, leading to episodes of AF (33). In our cohort, CRP was significantly elevated in patients in the AFDAS group. However, we did not further refine whether the duration of persistently elevated CRP led to a prolonged period of a single AF episode or an increased frequency of seizures, as a sustained inflammatory response can also lead to an increased risk of AF persistence and embolism (33, 34). Furthermore, in our study, NIHSS scores were significantly associated with AFDAS with a median of 10, similar to NIHSS scores (11–13.4) in previous studies (30, 35). One study found that patients with higher NIHSS scores also had more elevated CRP and a poorer prognosis for stroke (36).

Studies have shown that the incidence of AF in patients with IS increases with age (37). In the present study, the age of patients with AF was 69.69 ± 12.46, similar to previous studies (10, 11). We also found that AFDAS was significantly associated with CHF, which was the same as the results of previous studies (38, 39). Age and CHF severity increase the incidence of AFDAS (10, 38). A possible reason for this finding is that heart failure leads to increased atrial afterload, sustained atrial myocyte stretch, and arterial wall stress, resulting in electrical propagation abnormalities that promote the development and maintenance of AF (40). As a result, the risk of AF and recurrence rates are relatively high in this group of patients.

It is well known that BNP is an essential biomarker of heart failure and atrial disease. It is synthesized and released by the ventricular and atrial myocardium in states of myocardial strain or by the brain after IS. Its inclusion in our model is helpful. A study of AFDAS with 10-day, 3-month, and 6-month ECG monitoring of IS patients found that IS patients with elevated BNP ≥100 pg/mL could benefit more from extended outpatient ECG monitoring (41). In our study, BNP in the AFDAS group was 119.60 (66.25–238.70) pg/ml. However, there were some differences with other studies (42, 43), which may be related to differences in population, the severity of comorbid cardiovascular disease, and sample size. It is also possible that BNP levels are relatively low in patients with AF with short episode duration or low episode frequency compared to those with high-load AF (27). Interestingly, in a study of 300 AFDAS with a mean number of days of ECG monitoring of 6.78 days, BNP levels ≤131 pg/mL were considered to exclude delayed AF in stroke survivors (44), which may have led to a missed diagnosis. Therefore, further understanding of the relationship between AFDAS and BNP levels is necessary to give individualized prevention strategies.

Our study has several limitations. Firstly, this study was a retrospective collection of patients with AIS during hospitalization. The duration of ECG monitoring may have been limited, which may have underestimated the incidence of AFDAS during hospitalization. In our analysis, patients underwent 2–3 monitoring methods, and the incidence of AFDAS was close to that previously reported (21). Secondly, the higher mortality rate in the AFDAS group compared to the non-AFDAS group may be related to the combination of more factors, such as CHF, higher age, and previous AIS. These factors, in themselves, increase the risk of death. Whether AFDAS additionally increases the risk of adverse events in patients with IS needs further exploration. Thirdly, echocardiographic measurements were not recorded for the entire cohort. Some studies have suggested that left atrial size was associated with AF after stroke (10, 45). Fourthly, a test cohort is lacking in this study and testing of the model will be carried out in a subsequent prospective study. Finally, although our prediction model has a high predictive power, it needs to be combined with ECG in the comprehensive assessment of stroke patients during clinical application.

## Conclusion

The newly developed nomogram prediction model can help clinicians identify patients at high risk for AFDAS and help them prioritize longer and more intensive cardiac monitoring of patients in clinical practice.

## Data availability statement

The original contributions presented in the study are included in the article/Supplementary material, further inquiries can be directed to the corresponding author/s.

## Ethics statement

The studies involving human participants were reviewed and approved by Ethics Committee of Cangzhou Hospital of Integrative Medicine. Written informed consent for participation was not required for this study in accordance with the national legislation and the institutional requirements.

## Author contributions

MP, ZL, and LH designed and supervised the study. LS and NZ acquired the data. MP and ZL provided statistical analysis for the data, had full access to all of the data in the study, and were responsible for the integrity of the data and the accuracy of the data analysis. MP drafted the manuscript. ZL and LH critically revised the manuscript. All authors contributed to the article and approved the submitted version.

## Conflict of interest

The authors declare that the research was conducted in the absence of any commercial or financial relationships that could be construed as a potential conflict of interest.

## Publisher's note

All claims expressed in this article are solely those of the authors and do not necessarily represent those of their affiliated organizations, or those of the publisher, the editors and the reviewers. Any product that may be evaluated in this article, or claim that may be made by its manufacturer, is not guaranteed or endorsed by the publisher.
