# Supplementary material for: A nomogram for predicting atrial fibrillation detected after acute ischemic stroke
Source: Front Neurol. 2022 Oct 14;13:1005885. doi: 10.3389/fneur.2022.1005885 (PMC9614087; doi:10.3389/fneur.2022.1005885)
Supplement: Supplementary file 1 [file Data_Sheet_1.docx]

***Supplementary Online Content***

**Supplementary Table 1.** The R packages used in our study

**Supplementary Table 2.** Comparison of characteristics between the training and validation cohorts.

**Supplementary Table 3.** Incidence of atrial fibrillation and anticoagulation rates in different subgroups of the TOAST typology

**Supplementary Table 4.** Diagnostic performance of AFDAS' nomogram model in training and validation cohorts.

**Supplementary Table 1** The R packages used in our study.

| **Function** | **Packages** |
| --- | --- |
| least absolute shrinkage and selection operator (LASSO) regression | “glmnet” and “car” packages; |
| Forest plot | “forestplot” package; |
| Nomograms and calibration curves | “rms” and “Hmisc” packages; |
| Receiver operating characteristic curve | “pROC”, “ROCR” and “gplots” packages |
| Decision curve analysis and clinical impact curve | “rmda” package; |
| Net reclassification index (NRI) and Integrated discrimination index (IDI) | “nricens” and “PredictABEL” packages; |
| Kaplan-Meier curves | “survival”, “dplyr” and “survminer” packages |

**Supplementary Table 2** Comparison of characteristics between the training and validation cohorts.

| **Variables** | **All patients**  **（*n*=5332）** | **Training cohort（*n*=3173）** | **Validation cohort（*n*=2159）** | ***P*** |
| --- | --- | --- | --- | --- |
| **Gender, *n* (%)** |  |  |  | 0.180 |
| Male^§^ | 3480(65.3) | 2048(64.5) | 1432(66.3) |  |
| Female^§^ | 1852(34.7) | 1125(35.5) | 727(33.7) |  |
| Age (years)*^†^* | 62.93±12.55 | 63.14±12.83 | 62.61±12.11 | 0.128 |
| **Physiological data on admission** |  |  |  |  |
| Heart rates (beats/min)*^†^* | 105.05±10.18 | 104.87±10.10 | 105.31±10.31 | 0.126 |
| MAP (mm Hg)*^†^* | 94.54±6.44 | 94.44±6.08 | 94.69±6.92 | 0.161 |
| BMI (kg/m^2^)*^†^* | 21.11±1.98 | 21.08±1.95 | 21.06±1.94 | 0.166 |
| **Comorbidity, *n* (%)** |  |  |  |  |
| Hypertension^§^ | 946(17.7) | 560(17.6) | 386(17.9) | 0.829 |
| Coronary artery disease^§^ | 344(6.5) | 192(6.1) | 152(7.0) | 0.149 |
| Congestive heart failure^§^ | 426(8.0) | 258(8.1) | 168(7.8) | 0.644 |
| Diabetes mellitus^§^ | 700(13.1) | 442(13.9) | 258(11.9) | 0.036 |
| COPD^§^ | 448(8.4) | 279(8.8) | 169(7.8) | 0.212 |
| Hyperlipidemia^§^ | 1196(22.4) | 692(21.8) | 504(23.3) | 0.187 |
| Previous AIS/TIA^§^ | 714(13.4) | 421(13.3) | 293(13.6) | 0.750 |
| Hepatic insufficiency^§^ | 440(8.3) | 250(7.9) | 190(8.8) | 0.230 |
| Renal insufficiency^§^ | 576(10.8) | 331(10.4) | 245(11.3) | 0.290 |
| **Neuroimaging, *n* (%)** |  |  |  |  |
| Cortical infarction^§^ | 1751(32.8) | 1031(32.5) | 720(33.3) | 0.514 |
| Subcortical infarction^§^ | 2785(52.2) | 1680(52.9) | 1105(51.2) | 0.205 |
| Brainstem infarction^§^ | 924(17.3) | 542(17.1) | 382(17.7) | 0.562 |
| Cerebellar infarction^§^ | 693(13.0) | 401(12.6) | 292(13.5) | 0.344 |
| Multiple lesions of arterial territory^§^ | 1695(31.8) | 986(31.1) | 709(32.8) | 0.174 |
| **Stroke location, n (%)** |  |  |  | 0.155 |
| Left-sided^§^ | 2803(52.6) | 1682(53.0) | 1121(51.9) |  |
| Right-sided^§^ | 2028(38.0) | 1213(38.2) | 815(37.7) |  |
| Bilateral^§^ | 501(9.4) | 278(8.8) | 223(10.3) |  |
| **Subtype of stroke, n (%)** |  |  |  | 0.138 |
| Large-artery atherosclerosis^§^ | 4188(78.5) | 2492(78.5) | 1696(78.6) |  |
| Cardioembolism^§^ | 478(9.0) | 279(8.8) | 199(9.2) |  |
| Small-artery occlusion^§^ | 239(4.5) | 134(4.2) | 105(4.9) |  |
| Other determined etiology^§^ | 213(4.0) | 124(3.9) | 89(4.1) |  |
| Undetermined etiology^§^ | 214(4.0) | 144(4.5) | 70(3.2) |  |
| **Severity on admission** |  |  |  |  |
| NIHSS score^*^ | 8.00(5.00, 10.00) | 8.00(5.00, 10.00) | 8.00(6.00, 10.00) | 0.515 |
| GCS score^*^ | 9.00(7.00, 11.00) | 9.00(7.00, 11.00) | 9.00(7.00, 11.00) | 0.157 |
| **Laboratory tests** |  |  |  |  |
| White blood cell count (×10^9^/L)^*^ | 6.74(5.80, 8.55) | 6.74(5.80, 8.55) | 6.74(5.80, 8.55) | 0.957 |
| Hemoglobin (g/L)^*^ | 114.00(110.00, 117.00) | 114.00(110.00, 117.00) | 114.00(110.00, 117.00) | 0.394 |
| Platelet count (×10^9^/L)^*^ | 156.00(99.00, 165.00) | 156.00(99.00, 165.00) | 156.00(112.00, 165.00) | 0.756 |
| Red blood cell (×10^12^/L)^*^ | 4.03(3.42, 4.49) | 4.05(3.47, 4.49) | 3.99(3.36, 4.49) | 0.032 |
| Serum creatinine (μmol/L)^*^ | 80.44(72.91, 86.84) | 80.44(72.91, 86.72) | 80.44(72.91, 87.27) | 0.591 |
| Blood urea nitrogen (mmol/L)^*^ | 5.70(4.30, 6.81) | 5.70(4.30, 6.90) | 5.70(4.28, 6.81) | 0.409 |
| ALT (U/L)^*^ | 35.00(24.00, 46.00) | 35.00(24.00, 46.00) | 35.00(24.00, 46.00) | 0.594 |
| Bilirubin (μmol/L)^*^ | 11.80(8.50, 16.40) | 11.70(8.30, 16.50) | 11.90(8.80, 16.10) | 0.269 |
| Albumin (g/L)^*^ | 40.50(37.30, 41.60) | 40.50(37.30, 41.60) | 40.50(37.30, 41.60) | 0.808 |
| Cardiac troponin I (ng/mL)^*^ | 0.011(0.001, 0.161) | 0.010(0.001, 0.149) | 0.011(0.001, 0.195) | 0.361 |
| Creatine kinase (U/L)^*^ | 65.00(42.00, 103.00) | 65.00(42.00, 104.00) | 64.00(41.00, 99.00) | 0.335 |
| Triglyceride (mmol/L)^*^ | 1.28(0.92, 1.80) | 1.28(0.93, 1.82) | 1.26(0.92, 1.78) | 0.113 |
| Total cholesterol (mmol/L)^*^ | 3.97(3.27, 4.78) | 3.93(3.25, 4.75) | 4.03(3.30, 4.80) | 0.038 |
| HDL-C (mmol/L)^*^ | 1.10(0.90, 1.33) | 1.10(0.90, 1.30) | 1.13(0.92, 1.35) | 0.458 |
| LDL-C (mmol/L)^*^ | 2.18(1.68, 2.79) | 2.18(1.69, 2.78) | 2.21(1.68, 2.79) | 0.558 |
| BNP (pg/mL)^*^ | 94.57(80.17, 110.01) | 94.89(80.46, 110.05) | 93.97(79.53, 109.85) | 0.270 |
| Fibrinogen (g/L)^*^ | 3.70(2.97, 4.69) | 3.65(2.94, 4.71) | 3.75(3.02, 4.69) | 0.175 |
| APTT (s)^*^ | 36.90(33.90, 40.80) | 36.80(33.80, 40.70) | 37.1(34.00, 40.80) | 0.295 |
| PT (s)^*^ | 13.50(12.80, 14.80) | 13.50(12.90, 14.70) | 13.50(12.80, 14.90) | 0.098 |
| INR^*^ | 1.05(0.98, 1.18) | 1.05(0.98, 1.17) | 1.04(0.97, 1.18) | 0.043 |
| D-dimer (mg/L)^*^ | 1.05(0.44, 2.68) | 1.14(0.45, 2.69) | 0.96(0.43, 2.67) | 0.066 |
| Lactic acid(mmol/L)^*^ | 4.40(3.70, 5.10) | 4.40(3.70, 5.10) | 4.42(3.67, 5.14) | 0.301 |
| Procalcitonin (μg/L)^*^ | 0.18(0.13, 0.79) | 0.17(0.13, 0.77) | 0.19(0.13, 0.83) | 0.053 |
| CRP (mg/L)^*^ | 19.30(4.06, 58.97) | 21.80(4.09, 58.60) | 17.70(3.99, 59.20) | 0.667 |
| **Outcome, *n* (%)** |  |  |  |  |
| AFDAS^§^ | 384(7.2) | 225(7.1) | 159(7.4) | 0.705 |

*^†^* Normally distributed continuous variables are presented as means with standard deviations and analyzed by Student’ s t-test.

^*^ Non-normally distributed continuous variables are presented as medians with interquartile ranges and analyzed by non-parametric test.

^§^ Categorical variables are presented as frequencies with percentages and analyzed by Chi-square test or Fisher’ s exact test.

MAP, mean arterial pressure; BMI, body mass index; COPD, chronic obstructive pulmonary disease; AIS, acute ischemic stroke; TIA, transient ischemic attack; NIHSS, national institute of health stroke scale; GCS, glasgow coma scale; ALT, alanine aminotransferase; HDL-C, high-density lipoprotein cholesterol; LDL-C, low-density lipoprotein cholesterol; BNP, B-type natriuretic peptide; APTT, activeated partial thromboplasting time; PT, prothrombin time; INR, international normalized ratio; CRP, C-reaction protein;AFDAS,Atrial Fibrillation Detected After Stroke.

**Supplementary Table 3.** Incidence of atrial fibrillation and anticoagulation rates in different subgroups of the TOAST typology

| **Variable** | **Atrial fibrillation rate** | | |  | **Anticoagulation rate** | | |
| --- | --- | --- | --- | --- | --- | --- | --- |
|  | **All Patients** | **Training Cohort** | **Validation Cohort** |  | **All Patients** | **Training Cohort** | **Validation Cohort** |
| Large-artery atherosclerosis, *n*(%) | 317(5.95) | 186(5.86) | 131(6.07) |  | 313(81.5) | 182(80.89) | 131(82.39) |
| Cardioembolism, *n*(%) | 21(0.39) | 13(0.41) | 8(0.37) |  | 20(5.21) | 12(5.33) | 8(5.03) |
| Small-artery occlusion, *n*(%) | 18(0.34) | 11(0.35) | 7(0.32) |  | 17(4.43) | 11(4.89) | 6(3.77) |
| Other determined etiology, *n*(%) | 14(0.26) | 7(0.22) | 7(0.32) |  | 14(3.65) | 7(3.11) | 7(4.40) |
| Undetermined etiology, *n*(%) | 14(0.26) | 8(0.25) | 6(0.28) |  | 14(3.65) | 8(3.56) | 6(3.77) |

**Supplementary Table 4** Diagnostic performance of AFDAS' nomogram model in training and validation cohorts.

| **Variable** | **Model 1** | |  | **Model 2** | |
| --- | --- | --- | --- | --- | --- |
|  | **Training Cohort** | **Validation Cohort** |  | **Training Cohort** | **Validation Cohort** |
| AUROC | 0.815 (0.777-0.853) | 0.808 (0.770-0.847) |  | 0.846 (0.811-0.882) | 0.841 (0.804-0.877) |
| Sensitivity | 0.755 | 0.572 |  | 0.736 | 0.723 |
| Specificity | 0.730 | 0.897 |  | 0.829 | 0.823 |
| Cut-off value | 0.055 | 0.121 |  | 0.081 | 0.080 |
| False positive rate | 0.270 | 0.103 |  | 0.171 | 0.177 |
| False negative rate | 0.245 | 0.428 |  | 0.264 | 0.277 |

AUROC, area under the receiver operating characteristic curve.
